# An expansin-like protein expands forage cell walls and synergistically increases hydrolysis, digestibility and fermentation of livestock feeds by fibrolytic enzymes

**DOI:** 10.1371/journal.pone.0224381

**Published:** 2019-11-05

**Authors:** Andres A. Pech-Cervantes, Ibukun M. Ogunade, Yun Jiang, Muhammad Irfan, Kathy G. Arriola, Felipe X. Amaro, Claudio F. Gonzalez, Nicolas DiLorenzo, John J. Bromfield, Diwakar Vyas, Adegbola T. Adesogan

**Affiliations:** 1 Department of Animal Sciences, University of Florida, Gainesville, FL, United States of America; 2 Division of Food and Animal Science, Kentucky State University, Frankfort, KY, United States of America; 3 Department of Microbiology and Cell Science, Interdisciplinary Center for Biotechnology Research, University of Florida, Gainesville, FL, United States of America; United States Department of Agriculture, Agricultural Research Service, UNITED STATES

## Abstract

Bacterial expansin-like proteins have synergistically increased cellulose hydrolysis by cellulolytic enzymes during the initial stages of biofuel production, but they have not been tested on livestock feeds. The objectives of this study were to: isolate and express an expansin-like protein (**BsEXLX1**), to verify its disruptive activity (expansion) on cotton fibers by immunodetection (Experiment 1), and to determine the effect of dose, pH and temperature for BsEXLX1 and cellulase to synergistically hydrolyze filter paper (**FP**) and carboxymethyl cellulose (**CMC**) under laboratory (Experiment 2) and simulated ruminal (Experiment 3) conditions. In addition, we determined the ability of BsEXLX1 to synergistically increase hydrolysis of corn and bermudagrass silages by an exogenous fibrolytic enzyme (**EFE**) (Experiment 4) and how different doses of BsEXLX1 and EFE affect the gas production (**GP**), in vitro digestibility and fermentation of a diet for dairy cows (Experiment 5). In Experiment 1, immunofluorescence-based examination of cotton microfiber treated without or with recombinant expansin-like protein expressed from *Bacillus subtilis* (BsEXLX1) increased the surface area by > 100% compared to the untreated control. In Experiment 2, adding BsEXLX1 (100 μg/g FP) to cellulase (0.0148 FPU) increased release of reducing sugars compared to cellulase alone by more than 40% (P < 0.01) at optimal pH (4.0) and temperature (50°C) after 24 h. In Experiment 3 and 4, adding BsEXLX1 to cellulase or EFE, synergistically increased release of reducing sugars from FP, corn and bermudagrass silages under simulated ruminal conditions (pH 6.0, 39°C). In Experiment 5, increasing the concentration of BsEXLX1 linearly increased (P < 0.01) GP from fermentation of a diet for dairy cows by up to 17.8%. Synergistic effects between BsEXLX1 and EFE increased in vitro NDF digestibility of the diet by 23.3% compared to the control. In vitro digestibility of hemicellulose and butyrate concentration were linearly increased by BsEXLX1 compared to the control. This study demonstrated that BsEXLX1 can improve the efficacy of cellulase and EFE at hydrolyzing pure substrates and dairy cow feeds, respectively.

## Introduction

Forages are the main component of the diet of dairy cattle [1,2] and grazing beef cattle yet their high fiber concentrations limit their digestibility and ruminant productivity [3,4]. This problem is more pronounced in the tropics and subtropics where abundant warm-season forages are the main ruminant livestock feeds [5,6]. Tropical and subtropical grasses are usually lower in nutritional quality than temperate grasses because of their higher fiber concentrations and the presence of lignin [7]. To increase their digestibility, such forages are sometimes treated with exogenous fibrolytic enzymes (**EFE**) [8] that can degrade some of the recalcitrant components of plant cell walls [1,9]. Consequently, such enzymes have been added to dairy [4,10,11] and beef cattle [2,12] diets to increase fiber digestibility but the results have been equivocal [13,14].

Cellulose and hemicellulose, the major structural polysaccharides in plants [9], are treated by EFE containing cellulase and hemicellulase to hydrolyze the polymers into glucose and xylose, respectively [1]. While the efficacy of EFE at degrading cellulose and hemicellulose is consistent with pure substrates [15], inconsistent responses have been observed with forages due to various anatomical and biochemical constraints [1,3] that include poor substrate accessibility to enzymes, suboptimal cellulase: hemicellulase or enzyme: substrate ratios as well as cell-wall cross linkages with phenolic acids and lignin [3,9].

Cellulosic chains are tightly packed in micro-fibrils along with hemicellulose and lignin in plant cell walls [1,9,13]. The efficacy of EFE at degrading cellulose is dependent upon its accessibility to the substrate. With greater accessibility, cellulase can effectively hydrolyze cellulose into glucose [9], which can further be metabolized into volatile fatty acids (**VFA**), and microbial protein in the rumen [16]. In the present study, we explored the use of a non-hydrolytic expansin-like protein for increasing hydrolysis of cellulosic substrates and improving efficacy of the fibrolytic enzymes.

Expansin proteins are encoded by plants [17,18], whereas expansin-like proteins are encoded by bacteria [19,20] and fungi [21], in both cases they can loosen, expand or disrupt plant cell wall constituents such as cellulose and hemicellulose [15]. Although their mode of action is unknown, it is presumed that expansin-like proteins break into the crevices formed by the cell wall interlacing microfibers, triggering conformational change leading to enlargement of the crevices between the forage fibers and subsequently to expansion of the cell wall [22–24]. The disruptive activity of expansin-like proteins from *Bacillus subtilis* **(BsEXLX1**) is attributed to weakening of hydrogen bonds on the cellulose surface [22] leading to enlarged crevices or gaps that allow greater accessibility of cellulolytic enzymes within the cellulose matrix [22,23,25]. A meta-analysis conducted by Liu *et al*. [15] showed that expansins and expansin-like proteins of plant or bacterial origin, respectively, synergistically increased both the rate and extent of sugars by cellulases from cellulose-based substrates. However, most previous studies on expansins and expansin-like proteins were conducted to increase biofuel production. Whether such proteins can synergistically increase effects of fibrolytic enzymes on the hydrolysis and digestibility of ruminant feeds is unknown.

The objectives of this study were to: isolate and express an expansin-like protein (BsEXLX1), to verify its disruptive activity (expansion) on cotton fibers (Experiment 1) by immunodetection and to determine the effect of dose, pH and temperature for BsEXLX1 and cellulase to synergistically hydrolyze filter paper **(FP)** and carboxymethyl cellulose (**CMC**) under laboratory (Experiment 2) and simulated ruminal (Experiment 3) conditions. In addition, we determined the ability of BsEXLX1 to synergistically increase hydrolysis of corn and bermudagrass silages by an exogenous fibrolytic enzyme (EFE) (Experiment 4) and how different doses of BsEXLX1 and EFE affect the gas production **(GP),** in vitro digestibility and fermentation of a diet for dairy cows (Experiment 5). The hypothesis was that recombinant BsEXLX1 will expand sections of cellulose microfibers, synergistically increase hydrolysis of pure substrates by cellulase and that the combination of EFE and BsEXLX1 will increase hydrolysis, fermentation and in vitro digestibility of the ruminant forages and diet by exogenous fibrolytic enzymes.

## Materials and methods

### Isolation and expression of BsEXLX1

*Bacillus subtilis* strain UD1022 was used; the gene systematically annotated as *yoaJ* (accession number: WP_015383820.1) was PCR-amplified and cloned into the p15TV-LIC expression vector [26]. The primers used to amplify the *yoaJ* gene were: (F-5’TTGTATTTCCAGGGCATGAGTGCATTTGTTGGTATGG 3’; R 5’CAAGCTTCGTCATCATTATTCAGGAAACTGAACATGGCC 3’) as previously described [27,28]. The strain, *Escherichia coli* DH5α, was used as a host for cloning and to maintain the plasmids, whereas the strain BL21 was used to express BsEXLX1.

Genomic DNA and plasmids were isolated using the Qiagen DNeasy Blood and Tissue kit and Qiaprep Spin Miniprep kits (Qiagen, California), respectively, as described in the manufacturer’s protocols. The expression vector and *yoaJ* cloning and protein expression and purification were performed as described by Pagliai *et al*. [27]. Briefly, the cells were grown at 112*×g* in 2 L of Luria Bertani **(LB)** medium amended with 2.5 mM betaine and 1 M sorbitol at 37°C under aerobic conditions. Once the culture reached an optical density of (OD) _600_ of 0.50 nm, the culture was cooled to 17°C and the recombinant protein was induced using 0.5 mM of isopropyl β-D-1-thiogalactopyranoside. After 16 h, the cells were recovered by centrifugation (7600 × *g*, 4°C for 15 min) and the pellet suspended in binding buffer (500 mM sodium chloride (NaCl), 5% glycerol, 50 mM Tris pH 8.0, 5 mM imidazole, and 0.5 mM tris (2-chloroethyl) phosphate (TCEP). Subsequently, the cells were lysed using a French press, and the lysate centrifuged at 14,100*× g* for 30 min at 4°C. The cell-free extract was applied to a metal chelate affinity-column charged with Ni^2+^ (Qiagen, Hilden, Germany). The protein was eluted using 250 mM of imidazole in 50mM Tris (hydroxy-methyl) amino-methane (TRIS) at pH 8.0. Finally, the eluted protein was dialyzed against 500 mM NaCl, 5% glycerol, 50 mM TRIS pH 8.0, and 0.5 mM TCEP. The protein concentration was determined using a protein assay kit (Bio-Rad, Hercules, CA, USA). Protein purity was confirmed using 12% (w/w) sodium dodecyl sulfate-polyacrylamide gel electrophoresis (SDS-PAGE) followed by Coomassie Blue staining overnight (Bio-Rad, Hercules, CA, USA).

### Experiment 1: Immunodetection of disruption (expansion) of cellulose by bacterial expansin-like protein (BsEXLX1)

The disruption of cellulose by BsEXLX1 was examined by indirect immunofluorescence [29]. Briefly, cotton samples (6 mg of sterilized rolled cotton, CVS pharmacy, Rhode Island, USA) were incubated in triplicate for 1 h (pH 4 and 50°C) in a sodium-citrate buffer (final volume of 500 μL) without (Control) or with BsEXLX1 (300 μg/g) (N = 6). A second set of samples were incubated simultaneously and used as negative controls for epifluorescence (Total N = 12). The samples were centrifuged at 13,000 × *g* for 1 minute, and fibers were fixed using 4% paraformaldehyde (PFA) for 10 minutes at room temperature. Samples were rinsed using washing buffer (500 mM TRIS, pH 8.4 containing 0.15 M NaCl, + 0.1% (v/v) TritonX-100) twice for 10 min each. Subsequently, the fibers were incubated overnight at 4°C with 1 μL of mouse monoclonal anti-polyhistidine (Sigma, MO, USA) diluted in 300 μL of blocking buffer (500 mM Tris, pH 8.4 containing 1.5 mM NaCl, 1% bovine serum albumin and 0.1% (v/v) TritonX-100). The samples were rinsed in washing buffer three times and incubated for 1 h (37°C in the dark) with 1 μg mL-1 Alexa Fluor®488 (A-11001) labeled goat anti-mouse Immunoglobulin G (IgG) (Invitrogen, California, USA) diluted in 300 μL of blocking buffer. Finally, the samples were washed three times with 1 mL of washing buffer. Fibers were placed on clean microscope slides covered with a cover slip and observed with a Zeiss Axioplan 2 epifluorescence microscope (Zeiss, Jena, Germany). Digital images were obtained with an AxioCam MRm digital camera and analyzed using the AxioVision software (Zeiss) [29]. The fiber sections were selected based on the “bubble effect” caused during the expansion of fiber following a procedure previously reported [30]. In addition, we examined the presence of expansin protein in the expanded regions of triplicate control and BsEXLX1 samples. The relative expansion was estimated using the function “ruler” in AxioVision software, one representative fiber per sample (3 samples per treatment) was measured only in the regions in which lgG was detected, data collected in μm were converted to percentage comparing the original thickness with the expanded region.

### Experiment 2: Effect of expansin-like protein dose and various pH and temperature conditions on cellulose hydrolysis by BsEXLX1 and cellulase

A pilot study was conducted to examine the effects of pH (from 3 to 9) and temperature (from 30 to 50°C) on release of reducing sugars when purified cellulose (CMC) was treated with BsEXLX1 with or without cellulase. The best dose of BsEXLX1 (100 μg/g of substrate) from previous studies [19,20,25] and a low dose of cellulase (0.0148 FPU) were used. Treatments included 1) Control (Water only) 2) BsEXLX1 only (100 μg/g of substrate) 3) Cellulase only (0.0148 FPU) and 4) Cellulase + BsEXLX1. Initially, samples were tested at four different temperatures 30, 35, 40, 50°C (N = 48) achieved by adjusting the setting of a thermomixer incubator (Eppendorf, Germany); these temperatures were chosen based on the ranges that can occur during ensiling and in the rumen of ruminant livestock (up to 40°C)[31,32]. Samples were incubated in triplicate and the experiment was repeated three times. Subsequently, the optimal temperature for both BsEXLX1 and cellulase (50°C), were used to evaluate the effects of different initial pH achieved using 0.05 M of citrate buffers (pH 3, 4, 4.8, 5 and 6), 0.05 M sodium phosphate (pH 7) and 0.05 M Tris buffers (pH 8 and 9). Buffer solutions were chosen based on the optimum pKa (N = 96). Both experiments were repeated in three independent runs. The results are presented as supplemental information (S2 and S3 Figs). Likewise, the hydrolytic capacity of BsEXLX1 alone was examined by incubating different doses of BsEXLX1 (0, 100, 200, and 300 μg/g of cellulose) with filter paper (FP, Whatman paper # 1, General Electric, USA), carboxymethyl cellulose (CMC, Sigma Aldrich, St. Louis, MO) and cotton fibers (CVS pharmacy, Rhode Island, USA).

The existence of synergistic cellulose hydrolysis by cellulase and BsEXLX1 was examined by incubating filter paper (FP) with different doses of BsEXLX1 (0, 100, 200, and 300 μg/g of cellulose) with cellulase. Positive controls of Bovine serum albumin (**BSA**) were incubated (100 and 300 μg/g of cellulose) with cellulase because it can mask the enzymatic hydrolysis by preventing non-specific binding [24]. Samples were incubated for 0, 3, 6, 12 and 24 h at 50°C and pH 4.0. All treatments and time points were examined in triplicate and the whole experiment was repeated two times (total of 6 treatments + control with no enzyme, N = 210). Substrate and buffer blanks were used in each experiment. Control samples were dissolved in 125 μL of citrate buffer and 375 μL of double-distilled water and incubated (pH 4.8 and 50°C) in Eppendorf tubes a using thermomixer incubator under sterile conditions (Eppendorf, Germany) based on Liu et al. [15]. For the cellulase treatments, 100 μL of double-distilled water was replaced with 100 μL of citrate-phosphate buffer (pH 4.8) containing 0.0148 filter paper units (**FPU**) of purified cellulase (1–4 β-Endoglucanase E.C. 3.2.1.4, Sigma Aldrich, St. Louis, MO) using the method described by Adney and Baker [33]. This enzyme has a high linkage specificity for cellooligosaccharides particularly 1–4 β beta-D-glucosidic linkages in cellulose, lichenin and cereal beta-D-glucans[34]. To explore effects of combining cellulase with each BsEXLX1 dose, 200 μL of double-distilled water in the BsEXLX1 treatments described above were replaced with 100 μL of cellulase and 100 μL of BsEXLX1. One unit of enzyme activity was defined as the amount of enzyme that catalyzed the conversion of 1 micromole of substrate per minute. This enzyme was chosen because synergistic hydrolysis of cellulose by BsEXLX1 has been reported with cellulases and endoglucanases but not with hemicellulases [15]

Fresh purified BsEXLX1 was used in each experiment following the procedure previously explained. Prior to this experiment, protein concentrations of BsEXLX1 and cellulase were estimated using a BSA standard curve. Cellulose hydrolysis was measured by quantifying the release of reducing sugars using the 3, 5 dinitrosalicylic acid (**DNS**) method [33]. The absorbance was measured at 540 nm using a microplate reader (BioTek, Vermont, USA) and compared to a D-glucose standard curve. The amount of reducing sugar released was expressed as mg glucose equivalent/g substrate. The DNS method was chosen because it can detect a wide range of reducing sugars with a free carbonyl group including pentoses, hexoses, disaccharides (except sucrose), and oligosaccharides that could be produced during cleavage of cellulose and hemicellulose by fibrolytic enzymes.

### Experiment 3: Synergistic effects between BsEXLX1 and cellulase under simulated ruminal conditions

In this experiment, synergistic effects of BsEXLX1 and cellulase on hydrolysis of FP were examined under simulated ruminal conditions (pH 6.0, 39°C). Samples were prepared by adding filter paper (6 mg) to a 500 μL reaction mixture using four treatments 1) Blank without enzyme and BsEXLX1 2) BsEXLX1 only (100 μL BsEXLX1 per g of substrate) 3) Cellulase only (0.0148 FPU) and 4) Cellulase + BsEXLX1. Samples were incubated for 0, 3, 6, 12 and 24 h in triplicate in two runs; the pH of the reaction mixture was maintained at 6.0 and samples were incubated in the thermomixer at 39°C (N = 120). Treatments 1 and 2 were not included in the analysis because their absorbance values were zero and no sugars were released.

### Experiment 4: Synergistic improvement of corn and bermudagrass silage hydrolysis by BsEXLX1 and a commercial exogenous fibrolytic enzyme

The synergistic activity of BsEXLX1 with a commercial exogenous fibrolytic enzymatic preparation (**EFE**) for ruminants (Xylanase-plus, Du Pont, Wilmington, USA) was examined under ruminal conditions (pH 6.0 and 39°C for 12 h). According to previous screenings [34,35] Xylanase-plus has higher specificity towards 1–4 β-d-xylosidic linkages in xylans, β-d-glucosides, β-1,4-glycosidic bonds in cellulose with some mannanase side-activity. Whole-plant corn and bermudagrass silages were used as substrates because they are widely fed to dairy cows in the southeastern of United States. Representative samples of the substrates were dried at 60°C for 48 h, and ground to pass through a 1-mm screen using a Wiley mill (Arthur H. Thomas, Philadelphia, PA). The OM, CP, NDF, ADF, and hemicellulose concentrations of the corn (% of DM) and bermudagrass (% of DM) silages were 87.7, 9.6, 42.5, 28.4, and 14.1, and 81.9, 15.3, 59.8, 38.6, and 21.2, respectively (DM basis). Twenty milligrams of each forage were incubated for 12 h at 39°C in a 500 μL of reaction mixture prepared by adding 125 μL of 0.1 M citrate-phosphate buffer (pH 6.0) and 375 μL of double-distilled water. Similarly, reaction mixtures containing two doses EFE (0, and 2.3 mg/g substrate) and two doses of BsEXLX1 (0 and 136 μg/g substrate DM) were also prepared. The initial concentration of reducing sugars from corn (0.375± 0.1 mg/g DM) and bermudagrass silages (1.25± 0.5 mg/g DM) were used to correct the values for respective treatments. The efficacy of treatments at improving fiber hydrolysis was examined in quadruplicate in three independent runs, and reducing sugars released were estimated with the DNS method (DNS positive materials calculated on a glucose-equivalent basis). The application rate for the EFE was based on the manufacturer’s guidelines and previous studies [11,35,36]. Based on the results from a previous study [25], to maximize synergistic potential, the lowest dose of BsEXLX1 was kept equal to the amount of endoglucanase activity in EFE. The endoglucanase activity in the EFE was approximately 6% (0.138 mg/g DM) of the total dose of EFE (2.3 mg/g DM); hence, the lowest dose of BsEXLX1 was kept at 136 μg/g to maintain an approximate 1:1 ratio between BsEXLX1 dose and endoglucanase activity. The cellulase and xylanase activities were 3043 and 39940 μmol of sugar released/min per gram of EFE respectively.

### Experiment 5: Effect of BsEXLX1 and EFE on in vitro gas production, digestibility and fermentation of a diet for dairy cows

The synergistic activity of BsEXLX1 with a commercial EFE for ruminants (Xylanase-plus, Du Pont, Wilmington, USA) was evaluated using the 24-h *in vitro* batch culture technique [37] with a dairy cow total mixed ration (**TMR**) as substrate (0.50 g; Table 1). Treatments were arranged in a factorial design with 4 levels of BsEXLX1 (control = 0, Low = 138, Medium = 276, and High = 414 μg/g DM) and 2 levels of EFE (0, 2.3 mg/g DM). The amounts of BsEXLX1 and/or EFE corresponding to the respective application dose were diluted in 0.1 M sodium citrate buffer (pH 6) based on previous experiments and a previous study [35]. The levels of BsEXLX1 were added to maintain approximate ratios of 1:0, 1:1, 1:2 and 1:3 between endoglucanase activity of EFE and BsEXLX1 concentration.

**Table 1:** Ingredients and chemical composition of the total mixed ration used for the *in vitro* ruminal fermentation experiment (Experiment 5).

Ingredient	% DM
Corn silage	25.0
Bermudagrass silage	20.0
Brewer's grain	8.0
Corn meal	21.4
Citrus pulp	4.0
Soybean hulls	3.0
Canola meal	15.0
Mineral mix	3.60
Total	100
Chemical composition (%)	
Dry matter	45.3
Organic Matter	91.1
Ash	7.9
Crude Protein	16.3
Acid Detergent Fiber (ADF)	19.3
Neutral Detergent Fiber (NDF)	35.4
Hemicellulose^1^	16.1
NEL^3^ (Mcal/ kg)	1.60
NFC^2^	36.2

^1^Calculated as Hemicellulose = NDF- ADF

^2^Calculated as NFC = 100 –[CP + ash + fat + NDF]

^3^Net energy of lactation calculated from ingredient composition using values cited by Dairy NRC (2001)

The TMR substrate was weighed (0.5 g of DM; 1 mm; 91.1% OM) into previously dried F-57 Ankom bags (Ankom, Macedon, NY) after treatments were applied directly to the substrate. Bags were sealed using an electric sealer (Global industrial, USA) and immediately placed into 250 ml glass vials. Blank vials included in the assay consisted of only the bag and buffer, while controls contained only the substrate and buffer with no enzyme. Samples were incubated in triplicate and the experiment was repeated in three independent runs (n = 72).

Incubations were conducted using ruminal fluid collected from three lactating, ruminally-cannulated Holstein dairy cows mixed 2 to 3 h after consuming a similar TMR to the one that was incubated, which was comprised 20% bermudagrass haylage, 25% whole-plant whole-plant corn silage supplemented with a corn grain-based concentrate, and vitamins and minerals (NRC, 2001). The ruminal fluid was filtered through four layers of cheesecloth and mixed and buffered with pre-warmed artificial saliva (39°C) prior to dispensing into glass vials as described by Romero et al. [35,36] following approval of the protocol by the University of Florida Animal Care Research Committee. Buffered-ruminal fluid (52 ml) with an initial pH of 6.71± 0.2 was added to each vial containing bags with the substrate, and the vials were closed with rubber stoppers and crimped with aluminum seals.

Samples were incubated for 24 h at 39°C in a forced-air incubator and the samples were shaken during each gas measurement to ensure exposure of the substrates to the ruminal fluid. Bermudagrass silage samples were used as an internal standard to ensure in vitro digestibility values were in a normal range. Fermentation was terminated by placing the vial on ice. The total fermentation gas pressure within the vials was measured with a pressure transducer [38] and subsequently converted to gas volumes at 0, 3, 6, 12 and 24 h post-incubation. The following formula was used for conversion of gas pressure to volume in mL/ g OM incubated:

Gasvolume(mL)=(Gaspressure(psi)×4.8843)+3.1296

This equation was developed based on previous gas and pressure measurements conducted in our laboratory with the glass vials. After 24 h of incubation, 20 mL of headspace gas sample was collected and stored in vacuumed glass vials for methane analysis. Methane concentrations were estimated by gas chromatography (Agilent 7820A GC, Agilent Technologies, Palo Alto, CA) using a flame ionization detector and a capillary column (Plot Fused Silica 25 m × 0.32 mm, Coating Molsieve 5A, Varian CP7536). Temperatures for the injector, column and detector were maintained at 80°C, 160°C, and 200°C, respectively and N_2_ was used as carrier gas with a flow of 3.3 mL/min; the split ratio for CH_4_ in each sample was 100:1. Total methane concentrations were calculated in m*M*/L after 24 h. Methane emissions were expressed as m*M* per g of OM incubated. In addition, methane: VFA ratio was calculated by dividing total methane concentrations by total VFA concentrations.

After 24 h of incubation, filter bags containing the residues were rinsed with tap water several times and oven dried at 60°C for 48 h and weighed for quantifying DM digestibility. Dried residues were analyzed for neutral detergent fiber (**NDFD**) and acid detergent fiber (**ADFD**) digestibility [37] using an Ankom 200 Fiber Analyzer (Ankom, Macedon, NY). Hemicellulose concentration was estimated as the difference between the NDF and ADF concentrations and hemicellulose digestibility was calculated using hemicellulose concentrations and amounts of original and residual TMR before and after he 24 incubation. The pH of the residual buffered-ruminal fluid (initial pH was 6.71) in each tube was measured after 24 h of incubation (Accumet XL25 pH meter, Fisher Scientific, Pittsburgh, PA). The digestibility coefficients of the different fractions (NDF, ADF, and hemicellulose) were reported on an ash-free basis.

The residual inoculum in each vial was acidified with 50% H_2_SO_4_, and centrifuged at 8000 × *g* for 15 min at 4°C. The supernatant fluid was stored at -20°C and subsequently analyzed for VFA [39] using an HPLC system (Hitachi, L2400, Tokyo, Japan) fitted with a Bio-Rad Aminex HPX-87H column (Bio-Rad Laboratories, Hercules, CA).

### Statistical analyses

Experiment 1 data on BsEXLX1 disruption of cotton fibers were analyzed as a completely randomized design. Means were compared using the T-test at the 5% level of significance (Control vs BsEXLX1, three replicates per treatment + negative controls for immunofluorescence; n = 12).

Data from Experiment 2 on hydrolysis of pure substrates by cellulase and different doses of BsEXLX1 were analyzed as a randomized block design with 7 treatments (Control, Cellulase, Cellulase + 100, 200 or 300 μg/g of BsEXLX1 and Cellulase +100 or 300 μg/g of BSA) and 5 incubation periods (0, 3, 6, 12 and 24 h). The experiment was repeated in two independent runs and run was used as the blocking factor (n = 210). The following model was used:

$$Y_{ijk}=\mu +T_{i}+H_{j}+TH_{ij}+R{\left(random\right)}_{k}+e_{ijk}$$

Where μ = general mean, T_i_ = fixed effect of i^th^ treatment, H_j_ = fixed effect of j^th^ incubation period, (TH)_ij_ = interaction between i^th^ treatment and j^th^ hour of incubation, R_k_ = random effect of run k and e_ijk_ = experimental error.

Experiment 3 was analyzed as a randomized block design with a 2 × 2 × 5 factorial arrangement with 2 doses of cellulase (0, 0.014 FPU), 2 doses of BsEXLX1 (0, and 100 μg/g of BsEXLX1) and 5 incubation periods (0, 3, 6, 12, and 24 h. The experiment was repeated in two independent runs (N = 120). Data for Experiment 3 were analyzed using the following model:

$$Y_{ijk}=\mu +E_{i}+D_{j}+T_{k}+DE_{ij}+ET_{ik}+DT_{jk}+EDT_{\left(ijk\right)}+R{\left(random\right)}_{l}+e_{ijkl}$$

Where μ = general mean, E_i_ = fixed effect of i^th^ level of EFE, D_j_ = fixed effect of j^th^ level of BsEXLX1, T_k_ = fixed effect of k^th^ time of incubation, (ED)_ij_ = interaction between i^th^ level of EFE and j^th^ level of BsEXLX1, (ET)_ik =_ interaction between i^th^ level of EFE and k^th^ hour of incubation, (DT)_jk =_ interaction between j^th^ level of BsEXLX1 and the k^th^ hour of incubation, (EDT)_ijk_ = interaction between i^th^ level of EFE and j^th^ level of BsEXLX1, and the k^th^ hour of incubation R_k_ = random effect of run k and e_ijk_ = experimental error.

Experiment 4 was analyzed as a 2 × 2 × 2 factorial with two doses of EFE (0, 2.3 mg/g DM), two doses of BsEXLX1 (0, 136 μg/g DM) and two forages (corn silage; N = 48 and bermudagrass silage; N = 48). The treatments in Experiment 5 were arranged in a 2 × 4 factorial design with 2 levels of EFE (0, 2.3 mg/ g DM) and 4 levels of BsEXLX1 doses (0, 138, 276 and 414 μg/ g DM) in triplicate in three independent runs (N = 72). Experiments 4 and 5 were conducted in three independent runs and run was the blocking factor in the model.

In order to estimate the rate of fermentation (**K**_**d**_), Lag phase (**L**) and asymptotic gas production cumulative after 24 h (GP) in Experiment 5, the exponential model **Y = A (1−e**^**−c (t− l)**^**)** was used to estimate these parameters from each vial [40,41] where “Y” is the cumulative gas volume at time “t”, “A” is the potentially fermentable fraction “c” is fractional fermentation rate and “l” is the discrete lag phase. After calculating GP, K_d_ and L data were analyzed following the model:

$$Y_{ijk}=\mu +E_{i}+D_{j}+ED_{\left(ij\right)}+R{\left(random\right)}_{k}+e_{ijk}$$

Where μ = general mean, E_i_ = fixed effect of i^th^ level of EFE, D_j_ = fixed effect of j^th^ level of BsEXLX1, (ED)_ij_ = interaction between i^th^ level of EFE and j^th^ level of BsEXLX1, R_k_ = random effect k^th^ run and e_ijk_ = experimental error.

Before statistical analysis data from all experiments were explored for normality and homogeneity using Shapiro- Wilk test and Levene tests, respectively. For experiments from 2 to 5, means were compared using the Tukey test and significance was declared at P ≤ 0.05. Tendencies were declared at 0.05 ≤ P ≤ 0.10. When the effects of BsEXLX1 were significant in experiments 2 and 5, polynomial contrasts (linear and quadratic) were used to estimate the effect of the dose of BsEXLX1. The word synergy in this study was used to denote a statistically significant positive response resulting from an interaction of BsEXLX1 and EFE (P < 0.05), whereas, the term, antagonism was used for a statistically significant, and negative interaction. All data were analyzed using the package NMLE in R statistical program (RStudio 1.14, Boston, MA; http://www.rstudio.com/)

## Results and discussion

### Isolation and expression of BsEXLX1

Expansins and expansin-like proteins are non-hydrolytic proteins in plant cells and bacterial cells which play an important role in cell wall modification by loosening and disrupting hydrogen-bonds of cell wall polysaccharides during plant growth and elongation, whereas in bacteria, they promote root colonization [15,42]. The yield of the recombinant BsEXLX1 protein was 2.5 mg/L of culture broth. The yield was lower than those reported previously (10 mg/L) [15,25], but greater than levels of swollenins reported from *Trichoderma reesei* (0.025 mg/L in yeast;[15]). The molecular weight of the recombinant protein was ~27 kDa (S1 Fig and S5 Table), which is consistent with results observed in previous studies [17,25,43]. Previous studies have shown that expansin-like proteins from *Bacillus subtilis* share structural similarities with expansins secreted by plant cell walls like corn[15,25]; however, greater synergy between endoglucanases and cellulases has been reported with expansin-like proteins than with plant expansins [15].

### Experiment 1: Immunodetection of disruption (expansion) of cellulose by bacterial expansin-like protein (BsEXLX1)

Both plant expansins [42,43] and expansin-like proteins [19,25] possess no hydrolytic activity because they lack the typical catalytic domain found in common GH45 proteins[18,43]. In agreement with previous studies, no cellulose hydrolysis was detected when BsEXLX1 alone was incubated with CMC, filter paper, or cotton fibers (Table 2).

**Table 2:** Effects of different doses of a recombinant expansin-like protein BsEXLX1 on hydrolysis of cellulose from carboxymethyl cellulose (CMC), filter paper (FP), and cotton fiber substrates (Experiment 2; N = 3).

Substrate	BsEXLX1 dose, μg/g substrate				SEM	P-value
	0	100	200	300		
	Sugars released, mg/g substrate					
CMC (pH 4, 40°C)	0.074	0.075	0.074	0.075	0.002	0.49
FP # 1(pH 4, 50°C)	0.088	0.081	0.087	0.088	0.009	0.22
Cotton fibers (pH 4, 50°C)	0.086	0.087	0.088	0.087	0.001	0.72

**Fig 1:**
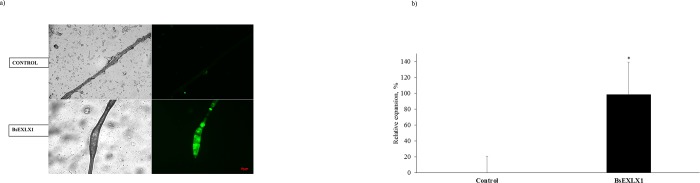
**a and b.** Effect of a recombinant bacterial expansin-like protein (BsEXLX1) on a) fluorescence microscopy images of cotton fibers treated with citrate buffer without (control) or with recombinant bacterial expansin-like protein (BsEXLX1) (300 μg/g cotton fibers) after incubation for 1 h in triplicate at 50°C and pH 4.0. Images on the left represent transmitted light illumination and, those on the right represent BsEXLX1 immunolocalization with a 20x objective. The scale bar represents 20 μm. b) Relative expansion of cotton fibers (expressed as a percentage of the value for the control). Samples were incubated in triplicate at 50°C and pH 4.0 for 1 hour. The concentration of BsEXLX1 was 300 μg/g of substrate. Symbol * denotes differences between treatments (P < 0.01; N = 3, Experiment 1).

Expansion of cotton fibers in response to BsEXLX1 was confirmed by microscopy (Fig 1A) and the size of the expanded sections of the treated cotton fibers was more than 100% greater compared to those of control samples (Fig 1B; P < 0.01). Despite the relative low frequency of expansions observed in the fibers (35±5% of positive expansions) expanded regions had a greater surface area compared to the control (Fig 1A). The expansion was attributed to BsEXLX1 because immunofluorescence revealed the presence of the recombinant BsEXLX1 protein in expanded sections of the cotton fibers (Fig 1A). Silveira and Skaf [22] showed expansion of cotton fibers in response to BsEXLX1 using computer simulation models; however, to the best of our knowledge, this study provides the first evidence of the disruptive nature of BsEXLX1 using immunofluorescence.

### Experiment 2: Effect of expansin-like protein dose and prevailing pH and temperature on cellulose hydrolysis by BsEXLX1 and cellulase

The efficacy of BsEXLX1 at expanding cell walls in experiment 1 led to investigation of its synergistic potential to enhance cellulase-mediated cellulose hydrolysis. Pilot studies were conducted to examine if synergy occurred over a wide pH and temperature range (S2 Fig and S3 Fig). Among temperatures, the enzymatic hydrolysis by cellulase was greater at 50°C compared with hydrolysis at 30, 35, and 40°C (P < 0.05). Similarly, synergistic cellulase—BsEXLX1 (100 μg/g of cellulose) activity at 35 and 50°C increased reducing sugar release by 50, and 8%, respectively (P < 0.05). Previous studies reported that 40, 45, and 50°C may be optimum temperatures for synergy between BsEXLX1 and Cellulase [15]. Cellulase activity, based on concentrations of reducing sugars released, was greatest at initial pH 4–4.8 and least at pH 6. Compared to cellulase only, synergistic cellulase-BsEXLX1 activity increased the amount of reducing sugar release by 34, 10, 48, and 54% at pH 4.0, 4.8, 5.0, and 6.0, respectively (P < 0.05). Carboxymethylcellulose is composed of ~60% cellulose and Cellulase + BsEXLX1 synergistically hydrolyzed 7.95, 7.05, 5.93, and 3.66% of the total cellulose available after 24 h, whereas Cellulase alone hydrolyzed 5.91, 6.43, 4 and 2.38% of the total cellulose after 24 h at pH 4.0, 4.8, 5.0, and 6.0, respectively. No enzymatic activity was observed at pH 3.0 and 9.0 and no synergistic effects were observed at pH 7.0, and 8.0 as previously reported [15,20,25]. Based on results from the pilot study, acidic pH (4.0–4.8) and a moderate to high temperature (40–50°C) are required to enhance synergy between a low dose of BsEXLX1 (100 μg/g cellulose) and cellulase (0.0148 FPU) as noted previously [19,20,25].

**Fig 2:**
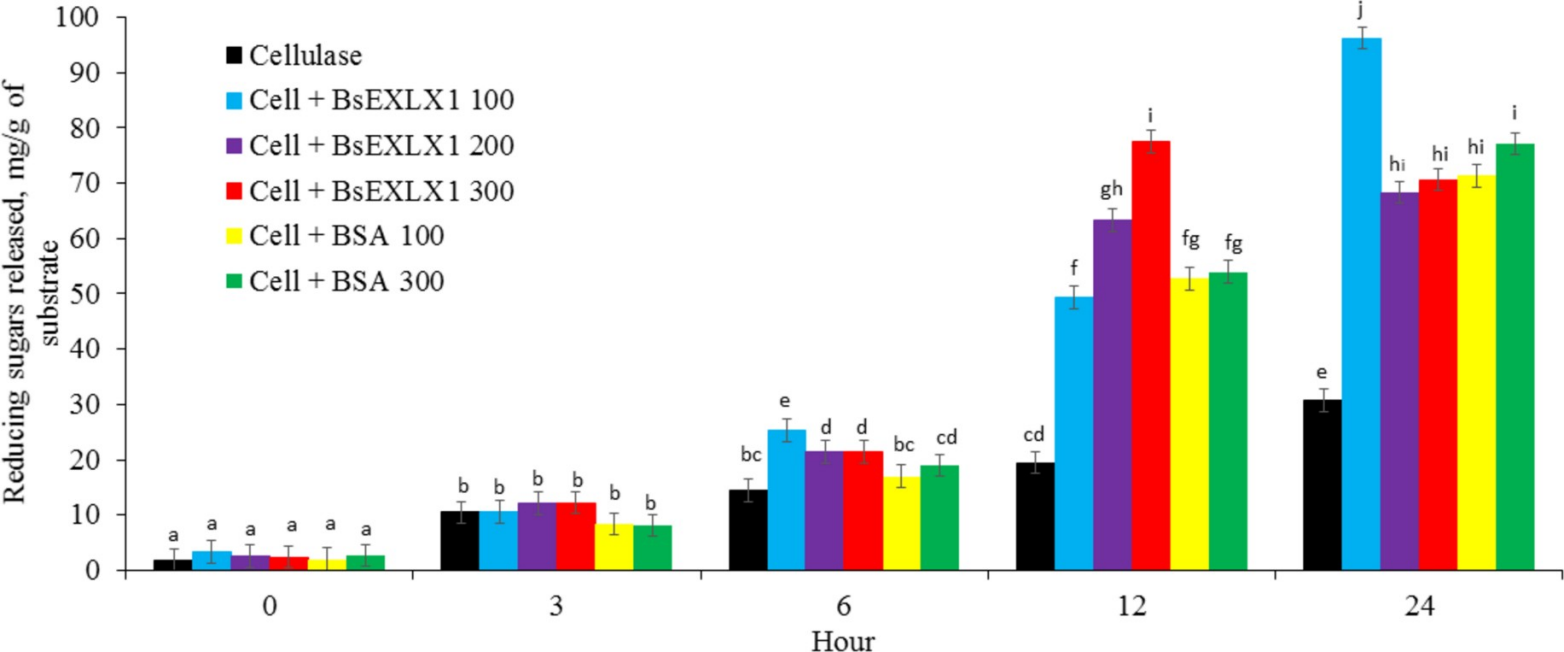
Effects of treatment with cellulase (0.0148 FPU) with or without different doses of bacterial expansin-like protein (BsEXLX1; 0, 100, 200, 300 μg/g) or two doses of BSA (100 and 300 μg/g) on hydrolysis of filter paper (pH 4.8; 50°C,) for different incubation periods (Treatment × Time interaction P < 0.01). Reducing sugars (DNS positive materials calculated on a glucose-equivalent basis materials). Different letters on bars indicate differences (P < 0.05) (Experiment 2).

When synergy between cellulase and different doses of BsEXLX1 (0, 100, 200, 300 μg/g cellulose) was examined at pH 4.8 and 50°C on FP (Fig 2), no treatment effects on reducing sugar release were detected after 0 or 3 h of incubation. However, non-specific binding by BSA was observed at 12 and 24 h, such that as expected, sugar release was overestimated. Bovine serum albumin (BSA) was used as a negative control to portray non-specific protein effects on cellulose hydrolysis that could lead to overestimation of the quantity of sugars released by the enzyme or BsEXLX1 [25]. Bunterngsook et al. [20] and Georgelis et al.[44] Reported similar non-specific binding with cellulose, causing an increase in reducing sugars; however, in agreement with this study, no sugars were released with BSA. Nevertheless, adding BsEXLX1 synergistically increased the hydrolytic activity of cellulase at 6, 12, and 24 h post-incubation compared to cellulase alone or the combination of cellulase and BSA (Fig 2).

The most effective dose of BsEXLX1 varied with the incubation duration. While 300 μg of BsEXLX1 was the most effective dose at increasing (35% more than BSA and cellulase) the amount of reducing sugars released by cellulase from filter paper after 12 h of incubation, 100 μg of BsEXLX1 was the most effective dose (42% more than BSA and cellulase) after 24 h of incubation. Kim et al. [25] reported a synergistic increase in sugar release (170%) by applying 100 μg of BsEXLX1 with cellulase (0.012 FPU/g) to filter paper in a citrate buffer solution (pH 4.8, 50°C) after 36 h of incubation. Likewise, Bunterngsook et al. [20] reported an increase in release of reducing sugars by 150, 30, and 1050% from avicel, phosphoric acid-swollen cellulose (PASC), and arabinoxylan, respectively, in response to adding bacterial expansins isolated from *Bacillus pumilus* (100 μg/g) and cellulase after 48 h of incubation. In contrast, Georgelis et al. [44] detected no synergistic effects of expansin-like proteins with cellulases when filter paper disks were used as substrates. The inconsistent responses between studies may be attributed to differences in substrate, enzyme and the different bacterial strains used for isolation of expansins. For instance, Georgelis et al.[44], who reported no synergy, used *Xanthomonas campestris*, *Ralstonia solanacearum* and *Clavibacter michiganensis*. However studies using *B*. *subtilis* strain 168 [25,45] and *B*. *subtilis* strain UD1022 (this study) detected synergistic hydrolysis between BsEXLX1 and cellulases. The inconsistent responses may also be attributed to differences in the expression systems such as those of *E*. *coli* vs *P*. *pastoris* [15], different doses of cellulase and or BsEXLX1, variations in substrate type (crystalline cellulose in avicel and CMC compared to insoluble cellulose in filter paper) and incubation period [15]. Some authors have suggested that cellulase and expansins compete for binding sites on the substrate [17,18,46], which could explain why the highest dose of BsEXLX1 decreased the synergistic increase in reducing sugar release after 24 hours.

### Experiment 3: Synergistic effects between BsEXLX1 and cellulase under simulated ruminal conditions

**Fig 3:**
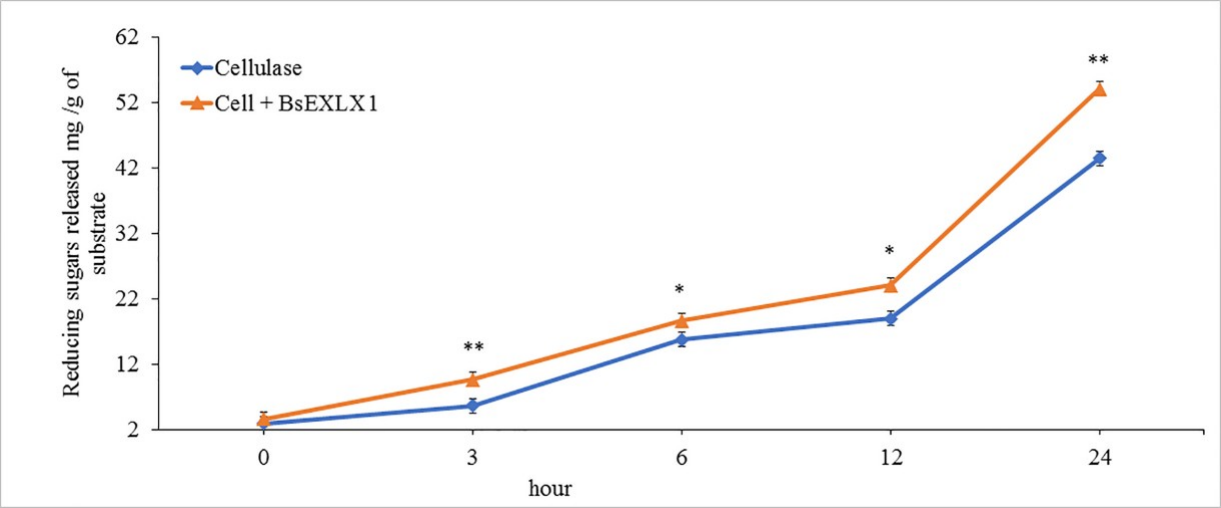
Synergistic effects of cellulase and bacterial expansin-like protein (BsEXLX1; μg/g) on filter paper (# 1) hydrolysis under simulated ruminal conditions of dairy cows (pH 6.0, 39°C, N = 4). Symbol * represents a tendency (P = 0.09) and ** indicates differences between treatments at the same incubation duration (Cellulase × BsEXLX1 × Hour; P < 0.01). Error bars indicate standard errors of the mean (Experiment 3, N = 4). Reducing sugars (DNS positive materials calculated on a glucose-equivalent basis).

The effects of pH and temperature on synergism between cellulases and BsEXLX1 have been well documented [15,25]. However, no studies have explored synergistic effects under ruminal conditions in dairy cows (pH 6 and 39°C). Experiment 3 showed that adding BsEXLX1 and cellulase synergistically increased (P < 0.01) hydrolysis of filter paper after 3 and 24 h of incubation (70.2 and 24.5%) and tended to increase hydrolysis after 6 and 12 h (18.5 and 22.5%; Fig 3). Filter paper is composed of a minimum 98% cellulose and Cellulase + BsEXLX1 synergistically hydrolyzed 0.41 and 0.92%, of the total cellulose available after 12 and 24 h, respectively, whereas Cellulase alone hydrolyzed 0.32 and 0.73% of the total substrate available after 12 and 24 h, respectively. The results from the present study are in agreement with previous studies using BsEXLX1 and a similar dose of Cellulase [25]. Based on these results, we hypothesized that BsEXLX1 could increase the efficacy of cellulases at hydrolyzing ruminant feeds and diets. Compared to a positive control of cellulase alone, addition of BsEXLX1 and cellulase synergistically increased release of sugars (Fig 3) by more than 20% after 24 h. Based on previous studies [35,36], increases in fiber hydrolysis by 10% could increase in vitro digestibility and fermentation of warm-season forages by 12% and 10% respectively [35]

### Experiment 4: Synergistic improvement of corn and bermudagrass silage hydrolysis by BsEXLX1 and a commercial exogenous fibrolytic enzyme

**Fig 4:**
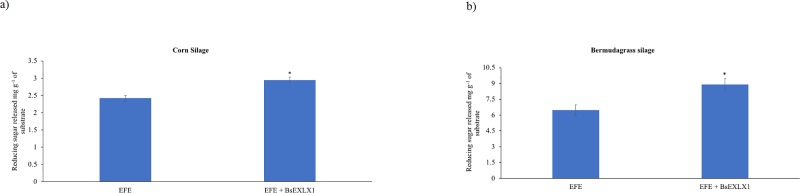
**a and b.** Synergistic hydrolytic effects of an exogenous fibrolytic enzyme (EFE) preparation and bacterial expansin-like protein (BsEXLX1; 100 μg/g DM) on a) whole-plant corn silage and b) bermudagrass silage under simulated ruminal conditions of dairy cows (pH 6.0, 39°C, 12 h, N = 4). Symbols on bars indicate differences (P < 0.05) between treatments (Experiment 4) the P-value for EFE× BsEXLX1 interaction was < 0.05. Reducing sugars were expressed as glucose equivalent/g substrate. Sugar values from control and BsEXLX1 only were equal to zero hence these were not presented.

Results of enzymatic hydrolysis of whole-plant corn silage and bermudagrass silage by EFE with or without BsEXLX1 are presented in Fig 4A and 4B, respectively. Compared to the blank, control samples and BsEXLX1 did not increase sugar release in either forages. Synergistic increases in hydrolysis of both forages were observed by combining BsEXLX1 and EFE treatments as reducing sugar yield was increased by 22 and 36%, respectively, compared to EFE alone (P < 0.05). To the best of our knowledge, this is the first documented evidence of synergistic potential of combining BsEXLX1 and cellulase at increasing hydrolysis of forages fed to ruminants. The greater (P < 0.01) synergy detected for bermudagrass silage compared to whole-plant corn silage may be due to the greater proportion of hemicellulose and cellulose in bermudagrass silage. Bunterngsook et al. [19] observed greater synergism between expansin-like proteins and fibrolytic enzymes in substrates containing a higher proportion of hemicellulose, probably due to breakage of hydrogen bonds between hemicellulose and cellulose, thereby increasing accessibility of cellulases to cell wall polysaccharides [21]. In addition, Bunterngsook et al.[19] Reported a 5-fold increase in cellulose hydrolysis when cellulases and expansin-like proteins were added to filter paper. The synergistic increases in forage cell wall hydrolysis observed in this study are promising. However, more studies are needed to examine whether synergistic improvement in fiber hydrolysis results in greater fiber digestibility and subsequently animal performance. In addition, we speculate that differences in the structure of cellulose may have contributed to variable responses between both substrates. Previous studies indicated that cellulose crystallinity is a rate determinant in enzyme-mediated hydrolysis as completely amorphous cellulose is hydrolyzed faster than crystalline cellulose from fungal enzymes (*Trichoderma reesi*) and ruminal fibrolytic bacteria like *Fibrobacter succinogenes* and *Ruminococcus flavifaciens* [47–50]. The greater synergy between BsEXLX1 and EFE with bermudagrass silage may be due differences in crystallinity between bermudagrass silage and corn silage [51,52].

### Experiment 5: Effect of BsEXLX1 and EFE on in vitro gas production, digestibility and fermentation of a diet for dairy cows

#### Gas production kinetics

The effects of a combination of BsEXLX1 and EFE on *in vitro* asymptotic gas production (GP), fermentation kinetics and in vitro digestibility of the TMR are summarized in Table 3. No interaction between BsEXLX1 and EFE was detected for GP, however addition of the EFE alone decreased the rate of gas production (K_d_; P < 0.01) and the lag phase (L) compared to the control (See S1 Table and S2 Table). Applying the highest dose of BsEXLX1 with EFE increased GP by 12.9% compared to EFE alone and by 27.5% compared to the control. Previous studies have shown lower fermentation rate (K_d_) and lag phase before fermentation (L) due to addition of EFE to warm-season forages like *Pennisetum purpureum* [53]. Jalilvand et al.[54] reported no effects of EFE addition on K_d_ and L of whole-plant corn silage, which is higher in NDF digestibility than bermudagrass silage. However, EFE addition to wheat straw, a low-quality forage, tended to increase L but did not affect K_d_ [54].

**Table 3:** Effect of a recombinant expansin-like protein (BsEXLX1) and EFE on asymptotic gas production (GP, mL/ g OM incubated) and in vitro digestibility after 24 hours of a total mixed ration for dairy cows (Experiment 5). Different letters in the same row indicate differences (P < 0.05). K_d_ was defined as rate of gas production (mL/h) and lag phase (L) was defined as the delay prior to the initiation of fermentation (h) (Experiment 5). Superscripts indicate L = linear effect of dose, Q = quadratic effect of dose (P < 0.05) and NS = no significant effect. In vitro digestibility values for NDF, ADF and hemicellulose (HEMD) were expressed in ash-free basis. Tables for main effects of EFE and BsEXLX1 is presented in S1 Table and S2 Table.

	Control				EFE					P- value		
BsEXLX1, μg/g DM	0	138	276	414	0	138	276	414	SEM	EFE	BsEXLX1	EFE × BsEXLX1
Asymptotic gas production, mL/ g OM	68.9	78.7	76.5	78.0	77.8	85.4	87.3	87.9	3.3	<0.01	0.01^(L)^	0.43
K_d_ (mL/h)	0.10	0.097	0.087	0.097	0.088	0.081	0.082	0.079	0.005	<0.01	0.21 ^(NS)^	0.67
Lag phase (h)	0.90	1.00	0.83	0.68	0.55	0.34	0.24	0.23	0.2	<0.01	0.53 ^(NS)^	0.85
DMD %	56.8	56.6	56.7	57.7	58.3	58.5	57.8	57.8	0.84	<0.01	0.88 ^(NS)^	0.45
OMD %	56.0	55.9	55.9	57.0	57.5	57.8	57.1	57.0	0.86	<0.01	0.88 ^(NS)^	0.45
NDFD %	31.3^de^	31.1^e^	32.3^cde^	34.1^bcde^	35.3^abc^	38.2^a^	34.9^abcd^	37.6^ab^	1.8	<0.01	0.01 ^(L)^	0.03
ADFD %	42.8^ab^	42.9^ab^	43.5^ab^	43.6^ab^	45.7^a^	45.7^a^	43.1^ab^	41.9^b^	1.3	0.12	0.15 ^(NS)^	<0.01
HEMD %	14.2	13.6	14.3	17.1	14.7	19.5	19.4	25.6	1.5	<0.01	<0.01 ^(Q)^	0.07

The TMR used in the present study contained a mixture of low- and high-quality forages and readily fermentable energy sources like corn grain, thus addition of the EFE probably caused hydrolysis and fermentation of soluble and insoluble fiber fractions [55], leading to a decrease in L. In a similar study, Adesogan et al. [40] reported no effects of EFE on fermentation GP, K_d_ and L of corn or bermudagrass silages. Previous studies in enzymology shown that bacterial expansin-like proteins from *B*. *pumilus* increased K_d_ under high concentrations of hemicellulose [20], in contrast BsEXLX1 from *B*. *subtilis* showed higher affinity towards lignocellulose than hemicellulose [45]. Therefore, we speculate that the high concentration of hemicellulose in the diet could explain the lack of effects in K_d_ in this study.

**Fig 5:**
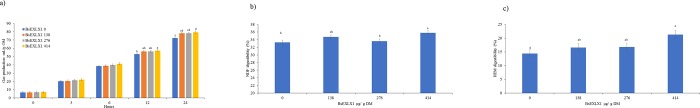
**a, b and, c.** Effect of dosing recombinant expansin-like protein (BsEXLX1) (μg/g DM) on a) cumulative gas production (mL/ g OM incubated) of a total mixed ration for dairy cows. b) In vitro digestibility of neutral detergent fiber (NDF) of a total mixed ration after 24 h incubation. c) In vitro digestibility of the hemicellulose fraction of a total mixed ration for dairy cows after 24 h incubation. Samples were incubated in triplicate and the experiment was repeated three times. Different letters on bars indicate differences (P < 0.05). For a and c polynomial contrasts showed a linear effect of increasing the dose in of BsEXLX1 (P < 0.01), whereas for b polynomial contrast showed a quadratic effect of increasing BsEXLX1. Error bars indicates SEM (Experiment 5).

Cumulative gas production was increased by addition of the highest dose of BsEXLX1 by 8 and 17.8%, respectively compared to the untreated control after 12 and 24 h incubation (Fig 5A). Considering that the highest dose of BsEXLX1 (0.414 mg) represents only 18% of the total dose of EFE (2.3 mg), results from study suggest that BsEXLX1 may be used to improve the efficacy of EFE preparations. However, more studies are required to confirm these findings.

#### Fiber digestibility

The increases in gas production by BsEXLX1 are probably due to greater NDF (Fig 5B) and hemicellulose (Fig 5C) digestibility (Table 3). Both GP and NDF digestibility linearly increased with increasing dose of BsEXLX1 after 24 h of incubation (P < 0.01). The effects on NDF digestibility and GP might be attributed to the greater accessibility or greater surface area of the cellulose fibers in response to BsEXLX1 addition, thereby allowing greater access for microbial enzymes to degrade cellulose fibers more rapidly and extensively [22]. The effects of BsEXLX1 on biofuel substrates have been well characterized [19,25,56]; however, this is the first study to evaluate effects of recombinant BsEXLX1 on fiber digestibility of a dairy cow diet. The increases in DMD and NDFD due to EFE addition agree with previous *in vitro* studies with commercial EFE containing cellulase and xylanase [35,36,57]. In this study, adding BsEXLX1 with the EFE synergistically increased (P = 0.03) NDF digestibility compared to the control. The magnitude of increase in NDF digestibility using a low dose of BsEXLX1 (138 μg/g DM) and EFE was 8.7% (P = 0.13) compared to EFE alone and 23.3% (P = 0.03) compared to the untreated control. Romero et al.[36] reported a 20% increase in NDF digestibility when bermudagrass haylage was treated with a high dose of EFE (4.6 mg/ g) compared to a control, which is similar to the 23.3% increase in NDF digestibility in the present study, though obtained using half of the dose of a similar EFE with BsEXLX1. These results suggest that BsEXLX1 had an enzyme-sparing effect as it increased fiber digestibility using a low dose of EFE. This reduction in the EFE dose could represent a cost saving for farmers provided they are not offset by the costs of the BsEXLX1.

The synergistic increase in NDF digestibility further validates our hypothesis that addition of recombinant BsEXLX1 will synergistically increase the hydrolytic capacity of cellulase or EFE on pure substrates and ruminant feeds.

Surprisingly, combination of the highest dose of BsEXLX1 and EFE had an antagonistic effect and decreased ADF digestibility (Table 3, P <0.01) compared to the EFE alone. The ADF fraction in forages contains mainly cellulose and lignin. Previous studies have suggested that when applied at high doses, BsEXLX1 competes for substrate binding sites with cellulases [18,25], possibility explaining why ADF digestibility decreased with the highest dose of BsEXLX1 and EFE.

Expansins may increase solubility of fibrous particles and water uptake as expansin-like proteins are chaotropic agents (like urea) and induce their effects by weakening the hydrophobic nature of cellulose fibers and reduces hydrophobicity[22]. Notably, increasing the dose of BsEXLX1 without EFE, increased hemicellulose digestibility of the diet compared to the untreated control (Fig 5C). The highest dose of BsEXLX1 increased hemicellulose digestibility by 18.7% more compared to the control (See S2 Table) implying that the expansin-like protein alone can be used to increase the digestibility and possibly the performance of ruminant livestock in the absence of EFE addition. This may be because disruption of cellulose and hemicellulose by BsEXLX1, increases accessibility of the endogenous ruminal and microbial fibrolytic enzymes within the lignocellulosic matrix of the diet. However, further research is required to validate these assumptions and simple and cheap methods for purification of the protein are required.

The expansion of the cell wall by BsEXLX1 would have increased the surface area available for microbial attachment and colonization. Previous studies have shown that expansin-like proteins promote root colonization by bacteria [23,56] (Fig 1A). Blocking the expression of the BsEXLX1 in *B*. *subtilis* reduces root colonization and fiber attachment by almost 100% [56]. Such a greater surface area and hence increased bacterial colonization probably explains how application of medium (276 μg/g DM) and high (414 μg/g DM) doses of BsEXLX1 alone increased hemicellulose digestibility. Future studies should examine effects of BsEXLX1 on ruminal microbial attachment and fermentation of dairy cow forages and diets.

The TMR used in the present study had higher concentrations of hemicellulose and non-fiber carbohydrates (including pectin and soluble fiber) than lignocellulose defined as acid detergent fiber (36.2 vs 19.3%). The highest dose (414 μg/g DM) of BsEXLX1 alone increased in vitro hemicellulose digestibility (Fig 5C, linear effect), perhaps reflecting greater affinity of bacterial expansin-like proteins like BsEXLX1 towards arabinoxylans, soluble glucans, and pectin [15,20,25].

#### VFA concentrations and methane emissions

**Fig 6:**
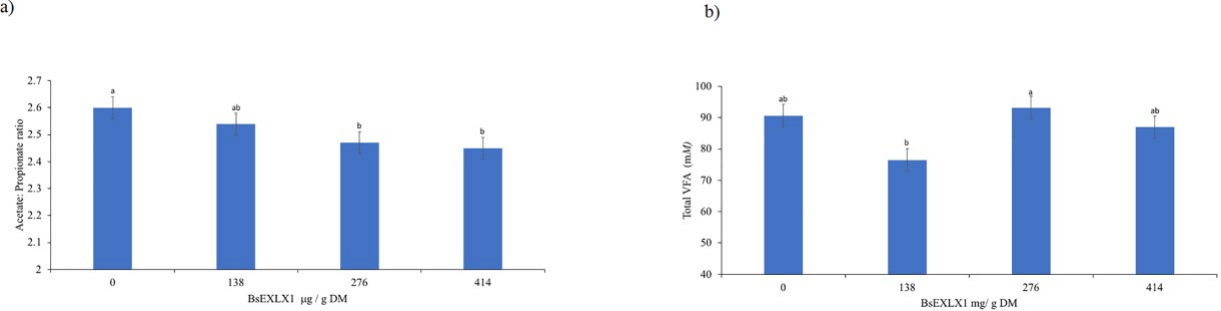
**a and b.** Effect of a recombinant expansin-like protein, BsEXLX1, on a) acetate: propionate ratio, polynomial contrast showed a linear effect of dosing BsEXLX1 (P < 0.01). b) Total VFA concentration of a diet for dairy cows. Different letters on bars indicate differences (P < 0.05), polynomial contrast showed a quadratic effect of dosing BsEXLX1 (P < 0.05). Error bars indicates SEM (Experiment 5).

The effects of the BsEXLX1 dose and EFE on fermentation parameters are summarized in Table 4. The combination of EFE and the lowest dose (138 μg/g DM) of BsEXLX1 tended to increase molar proportion of propionate compared to adding the EFE alone (20.9 vs 20.1%; P = 0.07). Therefore, addition of BsEXLX1 and EFE synergistically decreased the acetate: propionate ratio compared to control (P < 0.05). In contrast, no interactions were observed between EFE and BsEXLX1 on molar proportions of acetate; however, BsEXLX1 alone tended to decrease the molar proportion of acetate (P = 0.07) and linearly increased molar proportions of butyrate (P < 0.01). Consequently, increasing the dose of BsEXLX1 linearly decreased acetate: butyrate ratio compared to control. These results suggest that addition of BsELXLX1 to ruminant diets could improve energy conservation during fermentation of glucose to VFA, which increases net efficiency of ruminal digestion. This was evident from the greater molar propionate proportion when BSEXLX1 was combined with EFE and also from the lower molar proportion of acetate when high doses of BsEXLX1 alone were used (Fig 6A); however, the underlying mechanism is not clear and should be studied further in vivo.

**Table 4:** Effect of EFE and different doses of bacterial expansin-like protein (BsEXLX1) on *in vitro* volatile fatty acid concentrations (VFA) and total methane emissions, methane emissions per g OM incubated and total gas produced and CH_4_: VFA ratio from a diet for dairy cows. Samples were incubated in triplicate and the experiment was repeated three times. Different letters in the same row indicate differences (P < 0.05; Experiment 5). Superscripts indicate L = linear effect of dose, Q = quadratic effect of dose (P < 0.05) and NS = no significant effect. Tables for main effects of EFE and BsEXLX1 can be found at S3 Table and S4 Table.

	Control				EFE					P-value		
BsEXLX1 μg/g DM	0	138	276	414	0	138	276	414	SEM	EFE	BsEXLX1	EFE × BsEXLX1
Total VFA m*M*/L	95.1	73.2	98.2	87.3	84.6	79.7	87.3	85.8	5.0	0.26	<0.01^(Q)^	0.28
Individual VFA, mol/100 mol												
Acetate (A)	52.8	51.8	50.1	50.5	52.1	51.5	52.1	51.5	0.7	0.28	0.07 ^(L)^	0.13
Propionate (P)	20.2	19.9	21.1	21.2	20.1	20.9	20.4	20.5	0.4	0.52	0.22^(L)^	0.07
Butyrate (B)	13.1	13.5	13.8	13.8	13.3	13.8	13.9	13.8	0.2	0.44	<0.01^(L)^	0.69
A:P ratio	2.62^b^	2.61^b^	2.37^a^	2.38^a^	2.59^ab^	2.47^a^	2.56^ab^	2.52^a^	0.05	0.32	0.02^(L)^	0.01
B:P ratio	0.64	0.68	0.66	0.65	0.66	0.66	0.67	0.67	0.01	0.20	0.47^(NS)^	0.16
A:B ratio	4.07	3.83	3.60	3.67	3.91	3.75	3.77	3.74	0.08	0.97	<0.01^(L)^	0.16
Isobutyrate	2.2	1.5	2.3	1.9	1.7	1.6	1.9	1.9	0.2	0.17	0.03^(Q)^	0.47
Valerate	6.7	7.7	7.2	7.2	7.7	7.2	6.5	7.3	1.1	0.99	0.89^(NS)^	0.71
Isovalerate	4.9	5.3	5.2	5.2	4.9	4.9	5.1	4.9	0.2	0.04	0.28^(NS)^	0.42
Lactate m*M*/L	1.1	0	0	0.41	0.4	0	0.81	0.35	0.3	0.91	0.06^(Q)^	0.07
pH	6.58^b^	6.53^ab^	6.45^ab^	6.40^a^	6.41^ab^	6.44^ab^	6.46^ab^	6.44^ab^	0.04	0.07	0.31^(L)^	0.03
Methane emissions												
Total CH_4_ m*M*/L	11.0	10.7	10.6	10.3	10.4	10.4	10.7	10.6	0.32	0.48	0.61^(NS)^	0.23
CH_4_ m*M*/ g OM.	2.92	2.85	2.83	2.67	2.69	2.67	2.79	2.75	0.12	0.1	0.62^(NS)^	0.22
CH_4_: VFA ratio	0.118	0.154	0.112	0.120	0.125	0.132	0.124	0.126	0.008	0.94	0.01^(Q)^	0.16

Total VFA concentrations were not affected by EFE and no interactions were detected with BsEXLX1. However, the lowest dose of BsEXLX1 (138 μg/g DM) decreased VFA concentration (Fig 6A) while the medium (276 μg/g DM) and the highest (414 μg/g DM) doses did not have different effects from the control (quadratic effect, see S4 Table). The effects of BsEXLX1 on total VFA concentrations are difficult to explain because DMD and OMD were unaffected by BsEXLX1 even though NDFD and hemicellulose digestibility were increased when the highest dose of BsEXLX1 alone was added (Fig 5B and 5C). Previous studies have reported that addition of EFE increased digestibility of NDF without effects on VFA concentrations in warm-season grasses and whole-plant corn silage [36,58].We speculate that adding BsEXLX1 may have resulted in conversion of insoluble NDF to soluble NDF which may not be recovered through NDF filtration. Nevertheless, increasing soluble fiber may be beneficial as it reduces indigestible fiber (uNDF240) and hence reduces the bulk fill limitation to DMI. To overcome the apparent contradiction between digestibility and VFA responses, future studies should evaluate the effects of BsEXLX1 and EFE in under in vivo experimental conditions.

**Fig 7:**
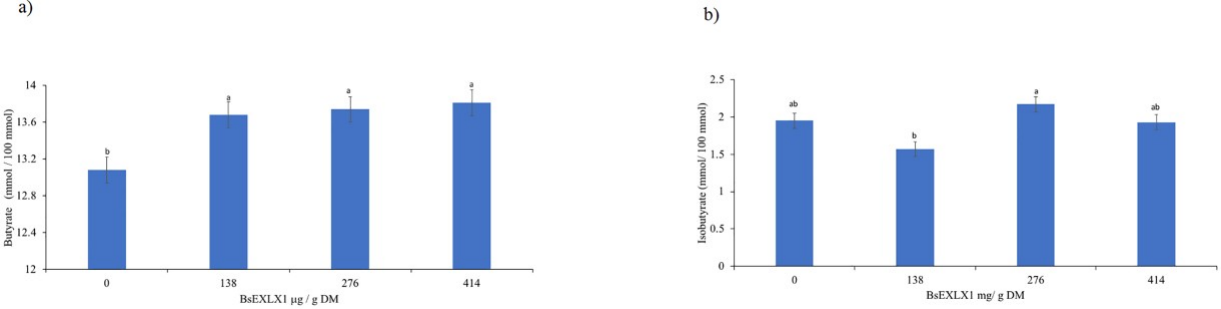
**a and b.** Effect of a recombinant expansin-like protein BsEXLX1 and EFE on a) butyrate, polynomial contrast showed a linear effect of dosing (P < 0.01) b) isobutyrate concentrations resulting from fermentation of a dairy cow diet, polynomial contrast showed a quadratic effect of dosing (P < 0.05). Different letters on bars indicate differences (P < 0.05). (Experiment 5).

Increasing the concentration of BsEXLX1 linearly increased molar proportion of butyrate and isobutyrate, compared to the Control (P < 0.01; Fig 7A and 7B); however, no interaction was observed with EFE (P = 0.69). Ruminal butyrate is an important substrate for milk fat synthesis in dairy cows [59] and because of its importance in rumen papillae development, ruminal butyrate is important for early lactation cows and calves. Future in vivo studies are required to test the effects of BsEXLX1 on VFA concentration in the rumen.

Despite increasing DMD, OMD, NDFD, and hemicellulose digestibility, EFE addition did not affect total VFA (P = 0.26) or VFA profile (P > 0.10) except for reducing molar proportion of isovalerate (P = 0.04). Previous studies have shown the potential of EFE to decrease acetate: propionate ratio [10,35,36], however some studies reported no effects [4,11]. These discrepancies may have been caused by variations among studies in enzyme and substrate type, and dose as well as enzyme activity.

Results from this study suggest that the highest dose of BsEXLX1 increased NDF (Fig 5B) and hemicellulose digestibility (Fig 5C) resulting in higher concentrations of butyrate (Fig 7A) and reducing acetate: propionate ratio (Fig 6A). Overall the addition of the highest dose of BsEXLX1 decreased the proportion of non-glucogenic acetate: butyrate compared to the control (3.99 vs 3.70, P <0.01). Future in vivo studies are required to test these findings.

Compared to the control, lactate concentrations tended to be lower when BsEXLX1 was added (P = 0.10), however the combination of EFE and the medium dose of BsEXLX1 (268 μg/g DM) tended to increase lactate concentrations compared EFE alone (0.4 vs 0.81 m*M*/L; P = 0.10). Nevertheless, lactate concentrations remained below 1.1 mM, therefore the effects detected in this study probably have relatively little biological relevance in cows.

Antagonistic effects between BsEXLX1 and EFE (P = 0.03) tended to decrease pH compared to the control, however compared to EFE alone, no differences were found. This effect could be explained by a greater H+ concentration due to a theoretical increase in NADPH resulting from the BsEXLX1-induced increase in butyrate concentrations and decrease in acetate proportions[60]. Previous research suggested that changes in pH in ruminal fluid can generally be attributed to changes in the concentrations of organic acids, feed digestibility and nutrient utilization [35,61]. In this study, despite increases in NDFD by synergy between BsEXLX1 and EFE, there were no effects on total VFA concentrations and pH. Similar discrepancies between digestibility, total VFA concentrations and pH levels were observed with EFE in previous studies [35,36,58] and the underlying reasons are not clear.

Despite differences in in vitro digestibility of NDF by synergistic action of EFE and BsEXLX1, total methane emissions did not differ between treatments in this study and no interactions were detected between EFE and BsEXLX1. However, compared to the Control addition of EFE tended to decrease methane emissions per gram of organic matter (2.82 vs 2.72 mM / g OM; P = 0.1). These results indicate that the intensity of methane production was lower in samples incubated with EFE compared to the control. While CH_4_ production has been increased in vitro and in vivo in some studies in response to addition of EFE to the diet [62,63], no response was observed in other studies [11,64]. In this study, addition of EFE did not change CH_4_: VFA ratio and no interactions were detected with BsEXLX1. Nevertheless, the lowest dose of BsEXLX1 alone increased the CH_4_: VFA ratio compared to control (0.14 vs 0.12; P = 0.01, quadratic effect of dosing). Previous studies with EFE have reported increases in digestibility, total VFA and CH_4_ concentrations after a long preincubation (at least 12 h) [62,64], however in this study neither EFE nor BsEXLX1 were preincubated; this may explain why total VFA and CH_4_ concentrations were not affected by EFE. Despite the lack of preincubation in this study, increasing the dose of BsEXLX1 linearly increased hemicellulose digestibility (Fig 5C), consequently molar proportions of butyrate linearly increased (Fig 7A), which could explain why the ratio of CH_4_: VFA was increased by dosing with BsXLX1 (see S4 Table). Future studies should examine effects of combining BsEXLX1 and EFE treatments on CH_4_ emission measurements to indicate how the combination affects greenhouse gas emissions.

## Conclusions

In conclusion, BsEXLX1, a 27 kDa protein produced by *B*. *subtilis*, disrupted or expanded the cell walls of cellulose fibers. The disruptive effect was attributed to the presence of added BsEXLX1 proteins in the regions of expansion, as confirmed by immunofluorescence. Despite the lack of detectable hydrolysis, adding BsEXLX1 alone to cotton fibers increased surface area and relative expansion. Furthermore, BsEXLX1 enhanced hydrolytic activity of a commercial EFE under simulated ruminal conditions (pH 6 and 39°C) as evidenced by greater yield of reducing sugars from whole-plant corn silage and bermudagrass silage. A synergistic increase in NDFD of a diet for dairy cows was detected when it was treated with BsEXLX1 compared to the control. Likewise, high and medium doses of BsEXLX1 alone increased in vitro hemicellulose digestibility of the same dairy cow diet. This study demonstrated that BsEXLX1 synergistically increased cellulase hydrolysis of various cellulose substrates. Similarly, EFE and BsEXLX1 synergistically increased hydrolysis and digestibility of ruminant feeds under laboratory and ruminal conditions. While the combination of BsEXLX1 and EFE appears promising for improving hydrolysis and digestibility of ruminant feeds, results should be interpreted with caution since these studies were conducted using purified substrates and forages under in vitro conditions. Future studies should test these findings under in vivo conditions.

## Supporting information

S1 Fig SDS-PAGE of proteins from recombinant *Escherichia coli*. Lanes: M, protein ladder with different masses; BsEXLX1, purified bacterial expansin-like protein (~27 kDa). Protein samples were separated by SDS-PAGE and stained with Coomassie Blue.
(TIFF)

S2 Fig Effect of the temperature on the synergistic hydrolysis of filter by a recombinant bacterial expansin-like protein (BsEXLX1) and cellulase. Samples were incubated in triplicate for 24 h at pH 4 and the experiment was repeated three times. Sugar release for control and BsEXLX1 alone were zero.
(TIF)

S3 Fig Effect of the pH on the synergistic hydrolysis of filter by a recombinant bacterial expansin-like protein (BsEXLX1) and cellulase. Samples were incubated in triplicate at 50°C for 24 h and the experiment was repeated three times. Sugar release for control and BsEXLX1 alone were zero.
(TIF)

S1 Table Effect of an exogenous fibrolytic enzyme preparation (EFE) on in vitro fermentation kinetics and digestibility of a total mixed ration for dairy cows. (DOCX)

S2 Table Effect of dose of a recombinant bacterial expansin-like protein (BsEXLX1) on in vitro fermentation kinetics and digestibility of a total mixed ration for dairy cows. (DOCX)

S3 Table Effect of an exogenous fibrolytic enzyme preparation (EFE) on in vitro fermentation products of a total mixed ration for dairy cows. (DOCX)

S4 Table Effect of dose of a recombinant bacterial expansin-like protein on in vitro fermentation products of a total mixed ration for dairy cows. (DOCX)

S5 Table Complete sequence, amino acid composition and chemical properties of recombinant BsEXLX1 (Yoaj gene) from *Bacillus subtilis* UD1022. (DOCX)
