# Supplementary figures and images for: An expansin-like protein expands forage cell walls and synergistically increases hydrolysis, digestibility and fermentation of livestock feeds by fibrolytic enzymes
Source: PLoS One. 2019 Nov 5;14(11):e0224381. doi: 10.1371/journal.pone.0224381 (PMC6830940; doi:10.1371/journal.pone.0224381)

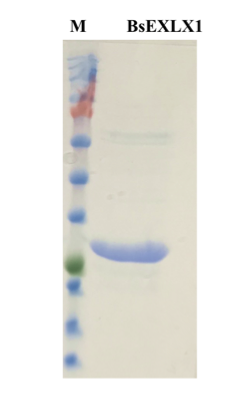

Supplement: S1 Fig — Lanes: M, protein ladder with different masses; BsEXLX1, purified bacterial expansin-like protein (~27 kDa). Protein samples were separated by SDS-PAGE and stained with Coomassie Blue. (TIFF) [file pone.0224381.s001.tiff]

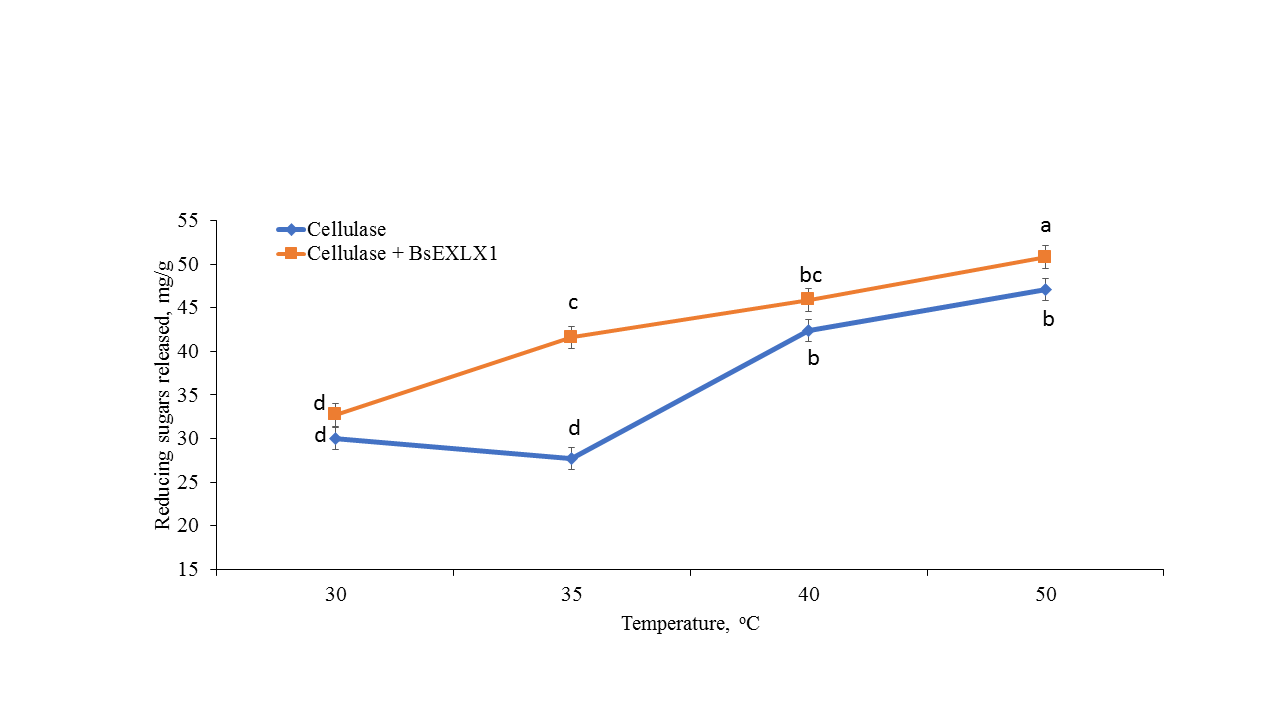

Supplement: S2 Fig — Samples were incubated in triplicate for 24 h at pH 4 and the experiment was repeated three times. Sugar release for control and BsEXLX1 alone were zero. (TIF) [file pone.0224381.s002.tif]

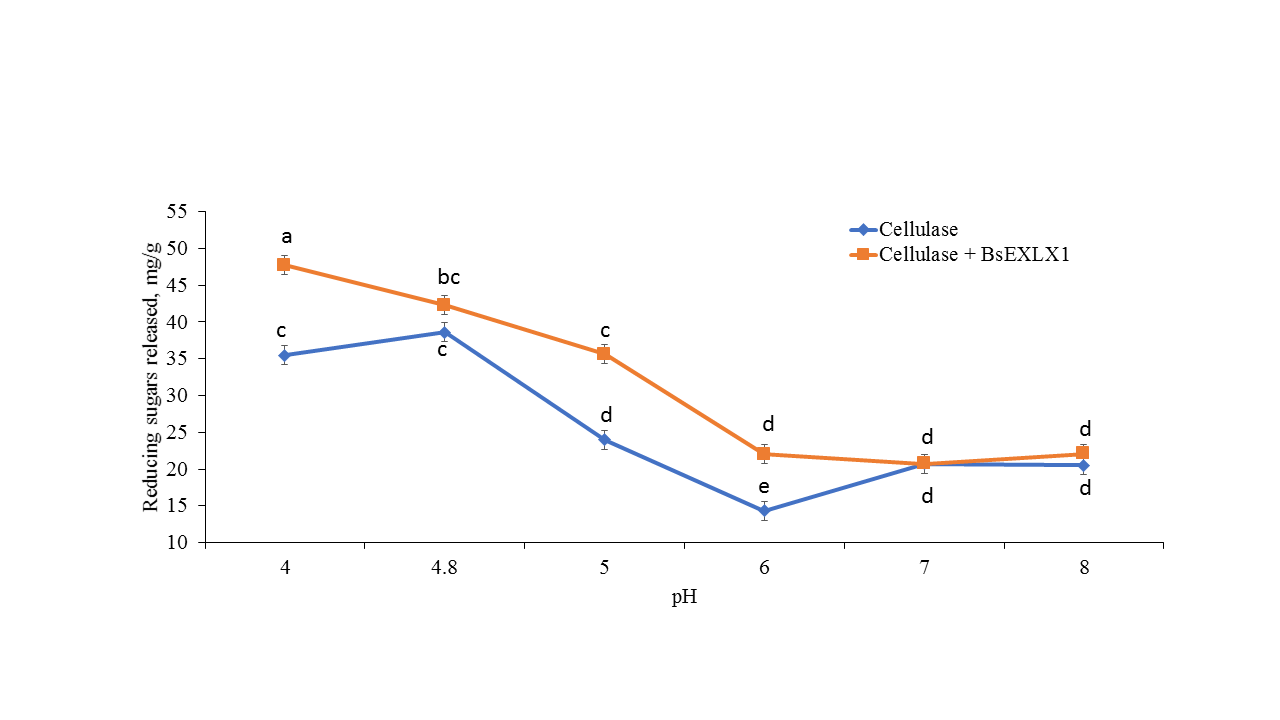

Supplement: S3 Fig — Samples were incubated in triplicate at 50°C for 24 h and the experiment was repeated three times. Sugar release for control and BsEXLX1 alone were zero. (TIF) [file pone.0224381.s003.tif]
