# Supplementary material for: An expansin-like protein expands forage cell walls and synergistically increases hydrolysis, digestibility and fermentation of livestock feeds by fibrolytic enzymes
Source: PLoS One. 2019 Nov 5;14(11):e0224381. doi: 10.1371/journal.pone.0224381 (PMC6830940; doi:10.1371/journal.pone.0224381)
Supplement: S1 Table — (DOCX) [file pone.0224381.s004.docx]

**S1 Table**

|  | Control | EFE | SEM | P-value |
| --- | --- | --- | --- | --- |
|  |  |  |  |  |
| Asymptotic gas production, mL/ g OM | 75.6 | 84.6 | 2.13 | <0.01 |
| Kd (mL/h) | 0.096 | 0.082 | 0.0003 | <0.01 |
| Lag phase (h) | 0.855 | 0.34 | 0.11 | <0.01 |
| DMD % | 57 | 58.1 | 0.66 | <0.01 |
| OMD % | 56.3 | 57.4 | 0.68 | <0.01 |
| NDFD % | 31 | 35.4 | 1.69 | <0.01 |
| ADFD % | 43.1 | 43.9 | 1.14 | 0.12 |
| HEMD % | 34.9 | 39.9 | 1.24 | <0.01 |
