# Supplementary material for: An expansin-like protein expands forage cell walls and synergistically increases hydrolysis, digestibility and fermentation of livestock feeds by fibrolytic enzymes
Source: PLoS One. 2019 Nov 5;14(11):e0224381. doi: 10.1371/journal.pone.0224381 (PMC6830940; doi:10.1371/journal.pone.0224381)
Supplement: S2 Table — (DOCX) [file pone.0224381.s005.docx]

| BsEXLX1, µg/g DM | 0 | 138 | 276 | 414 | SEM | BsEXLX1 | P-value | | |
| --- | --- | --- | --- | --- | --- | --- | --- | --- | --- |
|  |  |  |  |  |  |  | Linear | Quadratic | Cubic |
| Asymptotic gas production, mL/ g OM | 73.4^b^ | 82.1^a^ | 82^a^ | 83^a^ | 2.59 | <0.01 | 0.008 | 0.08 | 0.34 |
| Kd (mL/h) | 0.097 | 0.0892 | 0.0848 | 0.088 | 0.004 | 0.21 | 0.21 | 0.18 | 0.24 |
| Lag phase (h) | 0.727 | 0.673 | 0.537 | 0.462 | 0.15 | 0.53 | 0.3 | 0.94 | 0.29 |
| DMD % | 57.3 | 57.6 | 57.6 | 57.8 | 0.73 | 0.88 | 0.84 | 0.61 | 0.55 |
| OMD % | 56.6 | 56.8 | 56.9 | 57.1 | 0.74 | 0.88 | 0.844 | 0.61 | 0.55 |
| NDFD % | 32.1^b^ | 33.5^sb^ | 32.4^b^ | 34.7^s^ | 1.74 | 0.01 | 0.01 | 0.45 | 0.02 |
| ADFD % | 44.1 | 44.1 | 43.1 | 42.6 | 1.21 | 0.15 | 0.056 | 0.65 | 0.54 |
| HEMD % | 34.7^b^ | 37^ab^ | 36.9^ab^ | 41^a^ | 1.45 | <0.01 | 0.0002 | 0.39 | 0.18 |

**S2 Table**
