# Supplementary material for: An expansin-like protein expands forage cell walls and synergistically increases hydrolysis, digestibility and fermentation of livestock feeds by fibrolytic enzymes
Source: PLoS One. 2019 Nov 5;14(11):e0224381. doi: 10.1371/journal.pone.0224381 (PMC6830940; doi:10.1371/journal.pone.0224381)
Supplement: S3 Table — (DOCX) [file pone.0224381.s006.docx]

**S3 Table**

|  | Control | EFE | SEM | P-value |
| --- | --- | --- | --- | --- |
|  |  |  |  |  |
| Total VFA m*M*/L | 84.8 | 88.8 | 2.55 | 0.26 |
| Individual VFA, mol/100 mol | | |  |  |
| Acetate^1^ (A) | 51.4 | 51.8 | 0.58 | 0.28 |
| Propionate^1^ (P) | 20.6 | 20.5 | 0.35 | 0.52 |
| Butyrate^1^ (B) | 13.6 | 13.7 | 0.09 | 0.44 |
| A:P ratio | 2.5 | 2.54 | 0.02 | 0.32 |
| B:P ratio | 0.66 | 0.67 | 0.009 | 0.2 |
| A:B ratio | 3.79 | 3.79 | 0.04 | 0.97 |
| Isobutyrate^1^ | 2.02 | 1.82 | 0.11 | 0.17 |
| Valerate^1^ | 7.24 | 7.23 | 0.92 | 0.99 |
| Isovalerate^1^ | 5.18 | 4.97 | 0.16 | 0.04 |
| Lactate m*M*/L | 0.393 | 0.395 | 0.2 | 0.91 |
| pH | 6.5 | 6.44 | 0.02 | 0.07 |
| Total CH_4_ m*M*/L | 10.6 | 10.5 | 0.26 | 0.48 |
| CH_4_ m*M*/ g OM. | 2.51 | 2.54 | 0.04 | 0.39 |
| CH_4_: VFA ratio | 0.127 | 0.127 | 0.004 | 0.94 |
