# Supplementary material for: An expansin-like protein expands forage cell walls and synergistically increases hydrolysis, digestibility and fermentation of livestock feeds by fibrolytic enzymes
Source: PLoS One. 2019 Nov 5;14(11):e0224381. doi: 10.1371/journal.pone.0224381 (PMC6830940; doi:10.1371/journal.pone.0224381)
Supplement: S4 Table — (DOCX) [file pone.0224381.s007.docx]

**S4 Table**

| BsEXLX1, µg/g | 0 | 138 | 276 | 414 | SEM | BsEXLX1 | P-value | | |
| --- | --- | --- | --- | --- | --- | --- | --- | --- | --- |
|  |  |  |  |  |  |  | Linear | Quadratic | Cubic |
| Total VFA m*M*/L | 90.6 | 76.4 | 93.2 | 87 | 3.6 | <0.01 | 0.71 | 0.05 | 0.001 |
| Individual VFA, mol/100 mol | | |  |  |  |  |  |  |  |
| Acetate^1^ (A) | 52.5 | 51.7 | 51.1 | 51 | 0.66 | 0.13 | 0.01 | 0.39 | 0.87 |
| Propionate^1^ (P) | 20.2 | 20.4 | 20.7 | 20.8 | 0.39 | 0.22 | 0.04 | 0.76 | 0.79 |
| Butyrate^1^ (B) | 13.2^b^ | 13.7^a^ | 13.9^a^ | 13.8^a^ | 0.14 | <0.01 | 0.001 | 0.051 | 0.97 |
| A:P ratio | 2.61^a^ | 2.54^ab^ | 2.47^b^ | 2.45^b^ | 0.04 | 0.02 | 0.003 | 0.56 | 0.72 |
| B:P ratio | 0.65 | 0.67 | 0.67 | 0.66 | 0.01 | 0.47 | 0.55 | 0.15 | 0.79 |
| A:B ratio | 3.99^a^ | 3.79^b^ | 3.69^b^ | 3.7^b^ | 0.06 | <0.01 | 0.0003 | 0.056 | 0.92 |
| Isobutyrate^1^ | 1.98^ab^ | 1.57^b^ | 2.19^a^ | 1.95^ab^ | 0.15 | 0.03 | 0.43 | 0.04 | 0.006 |
| Valerate^1^ | 7.24 | 7.51 | 6.89 | 7.29 | 1.01 | 0.89 | 0.85 | 0.91 | 0.45 |
| Isovalerate^1^ | 4.92 | 5.12 | 5.19 | 5.07 | 0.17 | 0.28 | 0.26 | 0.11 | 0.87 |
| Lactate m*M*/L | 0.77 | 0 | 0.45 | 0.39 | 0.25 | 0.06 | 0.52 | 0.07 | 0.05 |
| pH | 6.5 | 6.49 | 6.46 | 6.43 | 0.03 | 0.31 | 0.06 | 0.71 | 0.96 |
| Total CH_4_ m*M*/L | 10.7 | 10.6 | 10.7 | 10.4 | 0.28 | 0.61 | 0.28 | 0.8 | 0.43 |
| CH_4_ m*M*/ g OM. | 2.56 | 2.52 | 2.53 | 2.5 | 0.05 | 0.45 | 0.27 | 0.91 | 0.65 |
| CH_4_: VFA ratio | 0.122^a^ | 0.143^b^ | 0.119^a^ | 0.123^a^ | 0.005 | 0.01 | 0.46 | 0.04 | 0.001 |
