# Supplementary material for: An expansin-like protein expands forage cell walls and synergistically increases hydrolysis, digestibility and fermentation of livestock feeds by fibrolytic enzymes
Source: PLoS One. 2019 Nov 5;14(11):e0224381. doi: 10.1371/journal.pone.0224381 (PMC6830940; doi:10.1371/journal.pone.0224381)
Supplement: S5 Table — (DOCX) [file pone.0224381.s008.docx]

**S5 Table**

| LYFQGMSAFVGMVLLTIFCFSPQASAAYDDLHEGYATYTGSGYSGGAFLLDPIPSDMEITAINPADLNYGGVKAALAGSYLEVEGPKGKTTVYVTDLYPEGARGALDLSPNAFRKIGNMKDGKINIKWRVVKAPITGNFTYRIKEGSSRWWAAIQVRNHKYPVMKMEYEKDGKWINMEKMDYNHFVSTNLGTGSLKVRMTDIRGKVVKDTIPKLPESGTSKAYTVPGHVQFPERSL |
| --- |

| Amino acid | Letter | Amount | Percentage |
| --- | --- | --- | --- |
| Ala | A | 18 | 7.60% |
| Arg | R | 9 | 3.80% |
| Asn | N | 10 | 4.20% |
| Asp | D | 12 | 5.10% |
| Cys | C | 1 | 0.40% |
| Gln | Q | 4 | 1.70% |
| Glu | E | 11 | 4.70% |
| Gly | G | 24 | 10.20% |
| His | H | 4 | 1.70% |
| Ile | I | 13 | 5.50% |
| Leu | L | 16 | 6.80% |
| Lys | K | 19 | 8.10% |
| Met | M | 9 | 3.80% |
| Phe | F | 9 | 3.80% |
| Pro | P | 13 | 5.50% |
| Ser | S | 15 | 6.40% |
| Thr | T | 15 | 6.40% |
| Trp | W | 4 | 1.70% |
| Tyr | Y | 14 | 5.90% |
| Val | V | 16 | 6.80% |
| Total aa |  | 236 |  |
| Weight |  | 26101.92 |  |
| Instability index | | 24.38 |  |
